# Accurate Prediction of Coronary Heart Disease for Patients With Hypertension From Electronic Health Records With Big Data and Machine-Learning Methods: Model Development and Performance Evaluation

**DOI:** 10.2196/17257

**Published:** 2020-07-06

**Authors:** Zhenzhen Du, Yujie Yang, Jing Zheng, Qi Li, Denan Lin, Ye Li, Jianping Fan, Wen Cheng, Xie-Hui Chen, Yunpeng Cai

**Affiliations:** 1 Shenzhen Institutes of Advanced Technology Chinese Academy of Sciences Shenzhen China; 2 Fiberhome Technologies College Wuhan Research Institute of Posts and Telecommunications Wuhan China; 3 University of Chinese Academy of Sciences Beijing China; 4 Shenzhen Health Information Center Shenzhen China; 5 FuWai Hospital Chinese Academy of Medical Sciences Shenzhen China

**Keywords:** coronary heart disease, machine learning, electronic health records, predictive algorithms, hypertension

## Abstract

**Background:**

Predictions of cardiovascular disease risks based on health records have long attracted broad research interests. Despite extensive efforts, the prediction accuracy has remained unsatisfactory. This raises the question as to whether the data insufficiency, statistical and machine-learning methods, or intrinsic noise have hindered the performance of previous approaches, and how these issues can be alleviated.

**Objective:**

Based on a large population of patients with hypertension in Shenzhen, China, we aimed to establish a high-precision coronary heart disease (CHD) prediction model through big data and machine-learning

**Methods:**

Data from a large cohort of 42,676 patients with hypertension, including 20,156 patients with CHD onset, were investigated from electronic health records (EHRs) 1-3 years prior to CHD onset (for CHD-positive cases) or during a disease-free follow-up period of more than 3 years (for CHD-negative cases). The population was divided evenly into independent training and test datasets. Various machine-learning methods were adopted on the training set to achieve high-accuracy prediction models and the results were compared with traditional statistical methods and well-known risk scales. Comparison analyses were performed to investigate the effects of training sample size, factor sets, and modeling approaches on the prediction performance.

**Results:**

An ensemble method, XGBoost, achieved high accuracy in predicting 3-year CHD onset for the independent test dataset with an area under the receiver operating characteristic curve (AUC) value of 0.943. Comparison analysis showed that nonlinear models (K-nearest neighbor AUC 0.908, random forest AUC 0.938) outperform linear models (logistic regression AUC 0.865) on the same datasets, and machine-learning methods significantly surpassed traditional risk scales or fixed models (eg, Framingham cardiovascular disease risk models). Further analyses revealed that using time-dependent features obtained from multiple records, including both statistical variables and changing-trend variables, helped to improve the performance compared to using only static features. Subpopulation analysis showed that the impact of feature design had a more significant effect on model accuracy than the population size. Marginal effect analysis showed that both traditional and EHR factors exhibited highly nonlinear characteristics with respect to the risk scores.

**Conclusions:**

We demonstrated that accurate risk prediction of CHD from EHRs is possible given a sufficiently large population of training data. Sophisticated machine-learning methods played an important role in tackling the heterogeneity and nonlinear nature of disease prediction. Moreover, accumulated EHR data over multiple time points provided additional features that were valuable for risk prediction. Our study highlights the importance of accumulating big data from EHRs for accurate disease predictions.

## Introduction

Cardiovascular diseases (CVDs) are currently the primary cause of global deaths according to a survey from the World Health Organization [1]. In 2016, 17.9 million people were estimated to have died of CVDs, representing 31% of all global deaths. Among these deaths, 85% are due to heart attack and stroke [2]. Modeling and prediction of CVD risk have long attracted the interest of many researchers. Several well-known risk scales such as the Framingham scales [3-5], American College of Cardiology/American Heart Association scales [6], QRISK [7], QRISK2 [8], and SCORE [9] have been established following years of population cohort studies, which provide an effective reference for clinicians to carry out disease prevention and treatment work [10].

Nevertheless, due to the complex and heterogeneous nature of CVD pathology, the prediction power of these risk scales has proven to be rather limited [11,12]. In recent years, researchers have been discovering or proposing new risk factors of CVDs according to lifestyle [13-15]; biochemical testing [16-18]; electrocardiograms [19-22]; medical imaging [23-28]; genetic, genomic, and proteomic biomarkers [29,30]; along with microbe and gene-environment interactions [31]. The steady growth of new emerging risk biomarkers surges demands for developing more precise disease prediction models. However, the traditional paradigm used for building risk models from a population-based study imposes a severe challenge to the development of accurate risk models, which usually requires a fixed set of observation variables at the beginning of the study and a lengthy follow-up period to collect all outcomes. Moreover, recent studies have identified that CVD risk factors vary according to social environments as well as ethnic and geographic differences [32,33]. This implies that an adaptive approach should be adopted for constructing more accurate CVD risk models that can be tuned to a specific population with higher efficiency.

Recently, the boosting of national or region-wide electronic health record (EHR) management systems has enabled the sharing and fusion of EHR data from many institutes [34], providing a faster approach for collecting large-scale population data to carry out retrospective cohort studies for more efficient assessments of CVD risk factors. A large-scale follow-up study using the EHR data of 1.25 million people identified the heterogeneous associations of blood pressure across different CVDs and age groups [35], which could not be discovered in previous population studies. Several efforts have also been made to create new disease risk prediction models based on EHR data using machine-learning models such as logistic regression, support vector machine (SVM), or K-nearest neighbor (KNN) approaches [36-39], but most of the results demonstrated very limited advantages compared with traditional risk scales. Compared with traditional cohort studies, EHR data are easier to acquire but the data quality is significantly inferior. Hence, one question that arises is whether EHR data are intrinsically unreliable and therefore unsuitable for achieving high-accuracy predictions. Moreover, studies on machine-learning approaches in EHR-based risk modeling are rather limited in the sense that almost all of the methods reported to date involve converting the EHR data into a single matrix, resulting in a lack of dynamic information. Therefore, establishment of a better modeling technique, more advanced machine-learning methods, and more data resources are expected to provide positive contribution to the power of existing prediction models.

Toward this end, the aim of the present study was to address these issues based on a case study using a large population of registered patients with hypertension in Shenzhen, China. Specifically, we evaluated the possibility of establishing a high-precision coronary heart disease (CHD) prediction model through big data and machine-learning methods. With a large population of 20,156 patients with CHD onset and more than 100 original features gathered from EHRs accumulated over 8 years, we were able to obtain more insight into risk factors than possible with traditional cohort studies, demonstrating that accurate prediction of CHD risks could be possible with the aid of large datasets, sophisticated machine-learning methods, and dynamic trends of patient information extracted from multiple time-point EHR records. These findings highlight the importance of accumulating EHR big data for accurate disease risk modeling, and provide a useful approach for the early screening and prevention of CVDs.

## Methods

### Overview of Sample and Data Processing

We investigated the stocked EHRs of registered patients with hypertension from the Shenzhen Health Information platform, which gathered the clinical records of 83 local public hospitals and over 600 community health service centers from 2010 to 2018. Each patient visiting the associated hospitals was assigned a unique identifier so that the clinical activities at multiple institutes could be merged. De-identification was performed on all data by the platform administrators under supervision of the Shenzhen Municipal Health Commission before collecting the datasets for investigation. Since all of the data were collected during regular clinical activities and were anonymized, following the Guidelines of the World Medical Association’s Declaration of Helsinki term 32, a waive-of-consent protocol was adopted, which was approved by the Shenzhen Institutes of Advanced Technology Institutional Review Board (No. SIAT-IRB-151115-H0084).

A total of 251,791 registered patients with hypertension were identified in the platform data. The collected EHR data for each patient included regular chronic disease follow-up records, inpatient and outpatient records, and clinical examinations and biochemical tests. Detailed field descriptions are provided in Multimedia Appendix 1. CHD diagnosis results were extracted from the main diagnosis field of the inpatient or outpatient records using the International Statistical Classification of Diseases and Related Health Problems (ICD)-10 [40] diagnostic codes I20 to I25 or the keywords related to CHD conditions, including “coronary heart disease,” “coronary sclerosis heart disease,” “ischemic cardiomyopathy,” “angina,” “acute myocardial infarction,” “myocardial ischemia,” “heart failure” (all translated from Chinese), and others, resulting in 37,776 cases of CHD onset.

To ensure the reliability of the outcomes, we required all samples to be associated with regular chronic disease follow-up information. A total of 23,335 samples were thus removed, resulting in 228,456 samples for analysis. We defined the follow-up period for each patient as the time interval between the most recent and the earliest record (regardless of record types) collected in the system. For positive samples (patients with CHD onset, n=33,279), we required the patient to be CHD free at the initial state and for the interval between the time of CHD diagnosis and the last CHD-free follow-up time to be within 0-3 years, which excluded 9027 patients, leaving 24,252 patients. Among the excluded patients, 9018 had a diagnosis of CHD onset but the diagnosis time was more than 3 years after the latest CHD-free follow-up. To avoid possible latencies in diagnosis, we excluded these patients from the present analysis, but the distribution of their prediction scores was analyzed later. For negative samples (non-CHD patients, n=195,177), we excluded 23,054 patients with other severe diseases (eg, death, stroke, cancer/tumor, renal failure, rheumatic heart disease, pulmonary heart disease, pericardial defect, heart valve disease, congestive heart failure, acute myocardial infarction) and 120,717 patients with a follow-up period less than 3 years, resulting in a set of 51,606 non-CHD samples. The reason for excluding patients with heart failure and myocardial infarction from the non-CHD set was that there may be a suspicion of CHD in such cases but without an explicit diagnosis. In addition, patients with other severe diseases would receive intensive medical interventions; thus, some of these patients may have previously had cardiac risks but interventions were administered prior to making a diagnosis of CHD. For example, the CHD risk scores of stroke patients without CHD were predicted to be high using our model (Multimedia Appendix 2); hence, these cases were excluded to avoid confusion. For positive samples, only the records during the CHD-free period were used for investigation. For negative samples, only the records from at least 3 years before the study endpoint were included. The recording time of the most recent included record for each patient was assigned as the baseline time point.

**Figure 1:**
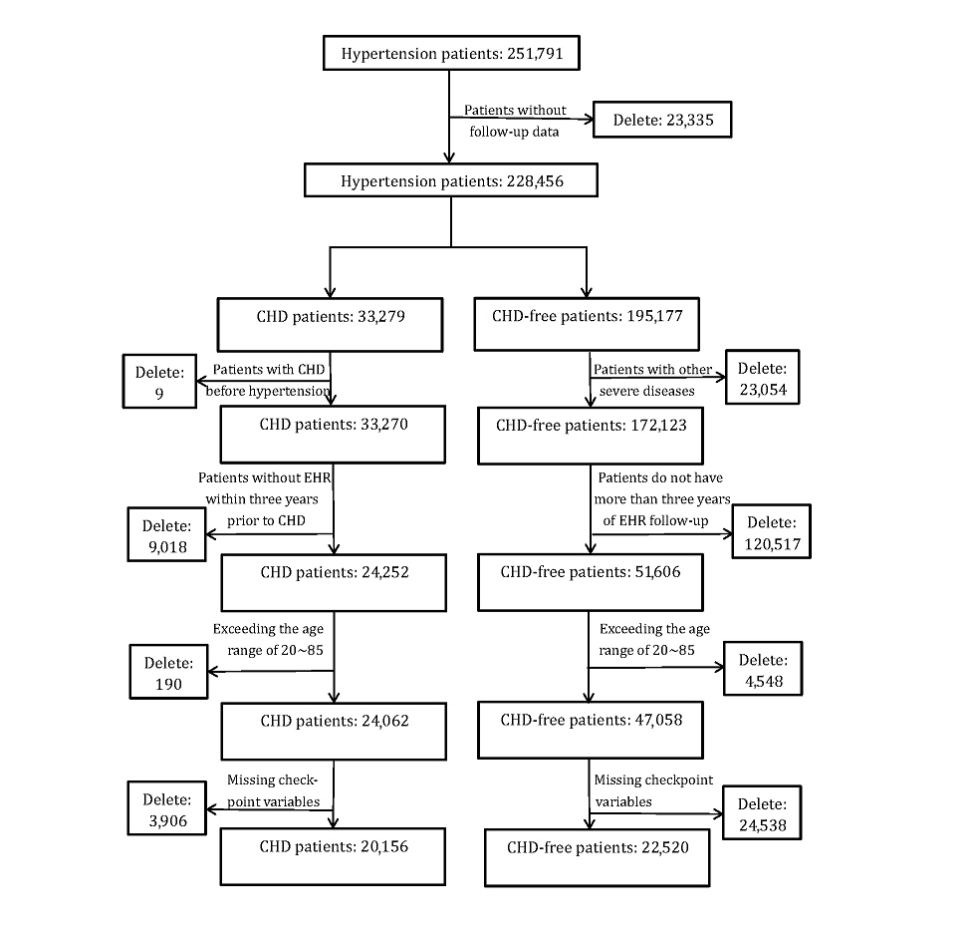
Patient cohort data processing. CHD: coronary heart disease; EHR: electronic health record.

EHR data usually contain abundant missing values. To avoid the influence of missing data on the prediction results, we used four basic variables as the quality filter of samples: age, gender, systolic blood pressure, and hypertension diagnosis time. Samples with no valid values for any of the above variables were excluded from the analysis. Moreover, only patients aged between 20 to 85 years were included in the study. Finally, we included data for 42,676 patients in the research cohort who met the above conditions, comprising 20,156 patients with CHD and 22,520 non-CHD patients. The above pipeline is schematically presented in Figure 1. Finally, the positive and negative samples were divided evenly to form the training set and the test set, respectively. Table 1 and Table 2 summarize the basic characteristics of both datasets. The distribution of the CHD-free time for the CHD group is shown in Multimedia Appendix 3.

**Table 1:** Sample distribution of the training and test datasets.

Subsample	Training set (N=21,338), n (%)	Test set (N=21,338), n (%)
Males	12,303 (57.66)	12,286 (57.58)
Females	9035 (42.34)	9052 (42.42)
Positive samples	10,078 (47.23)	10,078 (47.23)
Negative samples	11,260 (52.77)	11,260 (52.77)

**Table 2:** Basic characteristics of subjects for the two datasets.

Characteristic	Training set (N=21,338), mean (SD)	Test set (N=21,338), mean (SD)
Duration of illness (years)	5.8 (5.10)	5.7 (5.01)
Age (years)	49.97 (12.01)	49.52 (11.99)
Last SBP^a^ (mmHg)	131.39 (10.49)	131.37 (10.37)
Maximum SBP (mmHg)	135.40 (11.85)	135.70 (12.01)
Minimum SBP (mmHg)	128.21 (10.45)	127.96 (10.49)
Mean SBP (mmHg)	131.68 (9.66)	131.69 (9.57)

^a^SBP: systolic blood pressure.

### Feature Processing

In contrast to most existing research in the field, our dataset included multiple records with different record times for each patient. Therefore, data preprocessing and feature variable extraction, selection, and construction were crucial steps for the establishment and analysis of our model.

First, variables with over 20% missing values were removed from the study. Second, text parsing was performed. Inpatient and outpatient diagnostic results are a mixture of ICD codes and natural language text input. If the ICD codes were available for a record, we used the ICD codes directly as the annotation or features of the samples. Otherwise, by using an inhouse-designed lexical parsing code with keyword mapping and error corrections, we converted the diagnostic text into corresponding ICD codes. The parser was rule-based, in which each ICD code item was mapped to varied texts through a regular expression of keywords. The parsing procedure was carried out iteratively. At the end of each loop, the unparsed texts were collected and sorted by word frequency, and then a manual inspection was performed and the expressions were modified to match more text (including tolerating typographical errors). The loops continued until the unparsed texts were considered noninformative.

Third, accounting was carried out. Features from multiple sources (eg, examination, inpatient, and outpatient records) or multiple time points representing the same physiology index were gathered, and their maximum, minimum, or average values were calculated and used as new features. Fourth, for some rare diagnostic symptoms and similar symptoms (eg, diseases belonging to the same ICD class but less related to cardiac events) were merged into a single variable to avoid sparsity in value distribution. Finally, we divided the follow-up period of each patient into the early and late halves at the mid-time points. The frequency of specified events (eg, in-hospital or out-hospital visits, symptom onset) were accounted for each half, and the ratios were used as a new variable representing the trending status of the patients.

### Machine-Learning Algorithms

#### Extreme Gradient Boosting

Our model is based on the machine-learning algorithm XGBoost [41], which is short for extreme gradient boosting approach. XGBoost is an integrated machine-learning algorithm based on multiple decision trees with gradient boost as the framework. The loss function of XGBoost is defined as follows:

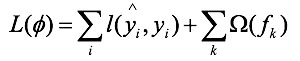



Where *l* is a differentiable convex loss function that measures the difference between the prediction *ŷi* and the target *y_i._* The second term Ω, as a regularization term, penalizes the complexity of the model. In contrast to the traditional gradient boosting decision tree method, XGBoost performs a second-order Taylor expansion on the loss function, and the additional regularization term helps to find the optimal solution for the whole, followed by weighing the decline of the objective function and the complexity of the model to avoid overfitting [41].

XGBoost supports missing values by default and naturally accepts a sparse feature format, allowing for directly feeding the data as a sparse matrix, and only contains nonmissing values (ie, features that are not presented in the sparse feature matrix are treated as “missing” and XGBoost will handle them internally). In tree algorithms, branch directions for missing values are learned during training. Internally, XGBoost treats nonpresence as a missing value and learns the best direction to handle missing values [41]. Equivalently, this can be viewed as automatically “learning” the best imputation values based on loss reduction. For continuous features, a missing (default) direction is learnt for missing value data to go into, so that missing data of a specific value will go in the default direction.

#### SVM

SVM is a generalized linear classifier that classifies data in a supervised learning manner, which was developed by Cortes and Vapnik [42]. The decision boundary is the maximum-margin hyperplane that solves the learning sample. The model trains a function that calculates a score for a new input to separate samples into two classes by building this hyperplane [43].

#### Logistic Regression

Logistic regression is a generalized linear regression analysis model [44], which is often used in data mining, automatic disease diagnosis, economic forecasting, and other broad applications. The algorithm is essentially a common two-category model, and the category corresponding to the object is obtained by inputting the attribute sequence of the object. The model assumes that the data obey the Bernoulli distribution, and uses the method of maximizing the likelihood function to solve the parameters with gradient descent to achieve the purpose of classifying the data.

#### Decision Tree

A decision tree algorithm is a method of building a model based on the characteristics of data using a tree structure [45]. A decision tree is usually composed of nodes and directed edges. The process of constructing decision trees usually includes feature selection, tree generation, and pruning. The essence of decision tree learning is to generalize a set of classification rules from the training dataset, representing a mapping relationship between object attributes and object values.

#### KNN

The KNN algorithm is used in the case where the data and labels are known in the given training set. The characteristics of the input test data are compared with the corresponding features of the training set to find the top *K* dataset most similar in the training set (ie, the most similar *K* instances, or nearest neighbors), and then the most frequently occurring classification among the *K* most similar data is summarized to classify the test data [46].

#### Random Forest

Random forest is an integrated learning algorithm that integrates multiple decision trees into a single classifier [47]. The random forest algorithm selects different splitting features and training samples to generate a forest of a large number of decision trees. When predicting unknown samples, each tree in the forest is made to make decisions, which improves the accuracy of the prediction compared to a single decision tree. By statistically determining the results of the decision, the classification with the highest number of votes is taken as the final classification result.

### Missing Data

For handling missing values in variables, XGBoost adopts an imputation-free approach in which missing values can be directly marked as “missing” in the input and the model can use only the nonmissing samples for creating trees, so that no value imputation operation was carried out. For the other algorithms, missing values were imputed with the average value of the entire population before model building.

### Implementation

All experiments were performed with the web-based interactive tool Jupyter notebook under the environment manager Anaconda, and a python3 kernel was used for data processing and modeling analysis. The XGBoost model relied on the “XGBClassifier” package, and the other machine-learning models were respectively dependent on the “LogisticRegression,” “svm,” “DecisionTreeClassifier,” “RandomForestClassifier,” and “KNeighborsClassifier” packages, which can be accessed from the sklearn library in the public Python software [48,49].

### Evaluation Criteria

We used a confusion matrix of the classification results to compute the performance indices, as shown in Table 3.

Based on this confusion matrix, we obtained the following indicators to evaluate the performance of our model. Accuracy was calculated as the proportion of the correct number of samples (true positives [TP]; the true category of the sample is positive and the final predicted result is also positive) to the total number of samples, including false negatives (FN; the true category of the sample is positive and the final predicted result is negative), TP, true negatives (TN; the true category of the sample is negative and the final predicted result is also negative), and false positives (FP; the true category of the sample is negative and the final predicted result is positive) using the following formula: TP+TN/TP+FP+TN+FN.

Sensitivity, also called recall, was calculated as the percentage of TP examples that were correctly predicted: TP/TP+FN.

The positive predictive value (PPV), also known as precision, was calculated as the percentage of positive samples that are predicted correctly: TP/TP+FP.

Specificity was calculated as the proportion of TN samples that was correctly predicted: TN/TN+FP.

The negative predictive value (NPV) was calculated as the percentage of the sample predicted correctly as a negative example: TN/TN+FN.

Finally, the F1-score was calculated as a harmonic average of model accuracy and recall according to the following formula: 2 × (precision×recall)/(precision+recall)

We then sorted the samples according to the prediction results of the model, and predicted the samples as positive examples one by one, successively obtaining the FP rate and TP rate, which were plotted as the horizontal and vertical coordinates to obtain the receiver operating characteristic curve (ROC). The area under the ROC value (AUC) was then selected as the main evaluation index. The more realistic meaning is that given a random positive and a negative sample, the probability of a positive sample output by the classifier is greater than that of negative sample output by the classifier. The formula for calculating AUC is as follows:

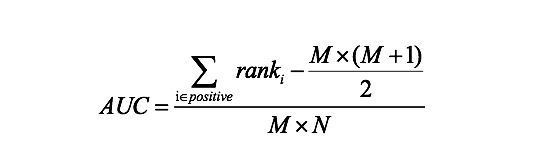



Where *M* represents the number of positive samples, *N* is the number of negative samples, and *rank_i_* is the order of probability from high to low for positive examples. Therefore, a larger AUC value indicates a better classification result of the learner and a better prediction effect of the model.

**Table 3:** Confusion matrix.

True_label	Predicted_label	
	Negative example (0)	Positive example (1)
Negative example (0)	True negative	False positive
Positive example (1)	False negative	True positive

## Results

### Model Prediction Performances

**Figure 2:**
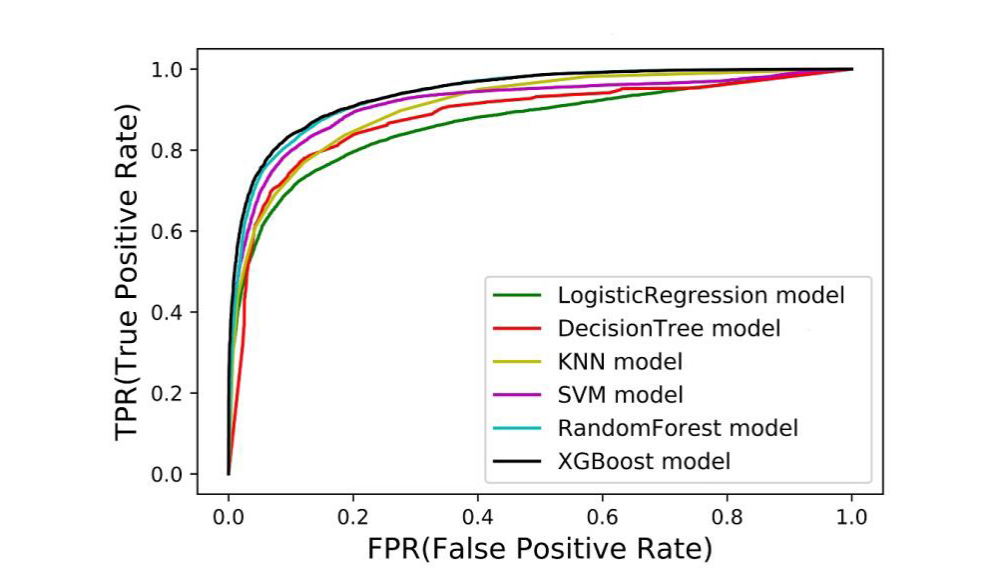
The receiver operating characteristic curves of models established by different algorithms. AUC: area under the curve.

After feature processing, a set of 65 feature variables were finally used as the input of the machine-learning algorithms. We conducted model training, verification, and prediction on the divided training set and test set. The prediction accuracy and AUC values of each model are shown in Table 4, and the detailed ROC curves are depicted in Figure 2. The nonlinear ensemble method XGBoost clearly achieved the highest accuracy on the test dataset. As a similar ensemble method, random forest achieved closely competitive performance. Machine-learning methods with nonlinear models (ie, random forest, KNN classifiers, decision trees, SVM) outperformed the traditional linear logistic regression model that has been widely used in most previous risk prediction models. This suggested that sophisticated machine-learning methods help to improve the performance of risk prediction with a sufficiently sized training dataset.

One potential concern would be that patients in the non-CHD group all had a total follow-up period of >3 years, whereas some patients in the CHD group may have had a follow-up period of less than 3 years, which would likely result in an inherent imbalance between the two groups of data. To exclude the possible bias introduced by variation in the total follow-up time, we carried out an additional experiment in which the test sets were divided into two groups: (1) CHD onset within 3 years and total follow-up >3 years (5094 samples), and (2) CHD onset within 3 years and total follow-up ≤3 years (4984 samples). We applied the same derived prediction model on these two test sets separately, which confirmed that the performance of the model was analogous on both sets with similar AUC values (0.9464 for group 1 vs 0.9389 for group 2; Multimedia Appendix 4) and there was no statistically significant difference on the risk score distributions between the two groups (*P*=.34 Kolmogorov-Smirnov test). This suggest that the inclusion of CHD patients with under a 3-year follow-up time did not introduce observable data bias and the models developed would be reliable in terms of generalization.

**Table 4:** Prediction scores of models created by different algorithms.

Algorithm/ model	AUC^a^	ACC^b^	F1-score	Sensitivity	PPV^c^	Specificity	NPV^d^
Logistic regression	0.865	0.809	0.785	0.736	0.840	0.874	0.787
Decision tree	0.882	0.827	0.802	0.742	0.873	0.903	0.796
KNN^e^	0.908	0.827	0.808	0.769	0.851	0.879	0.810
SVM^f^	0.915	0.850	0.832	0.782	0.888	0.912	0.824
Random forest	0.938	0.861	0.846	0.812	0.884	0.905	0.843
XGBoost	0.943	0.870	0.855	0.820	0.895	0.914	0.849

^a^AUC: area under the receiver operating curve.

^b^ACC: accuracy.

^c^PPV: positive predictive value.

^d^NPV: negative predictive value.

^e^KNN: K-nearest neighbor.

^f^SVM: support vector machine.

### Contributions of EHR Feature Variables to Model Prediction

**Figure 3:**
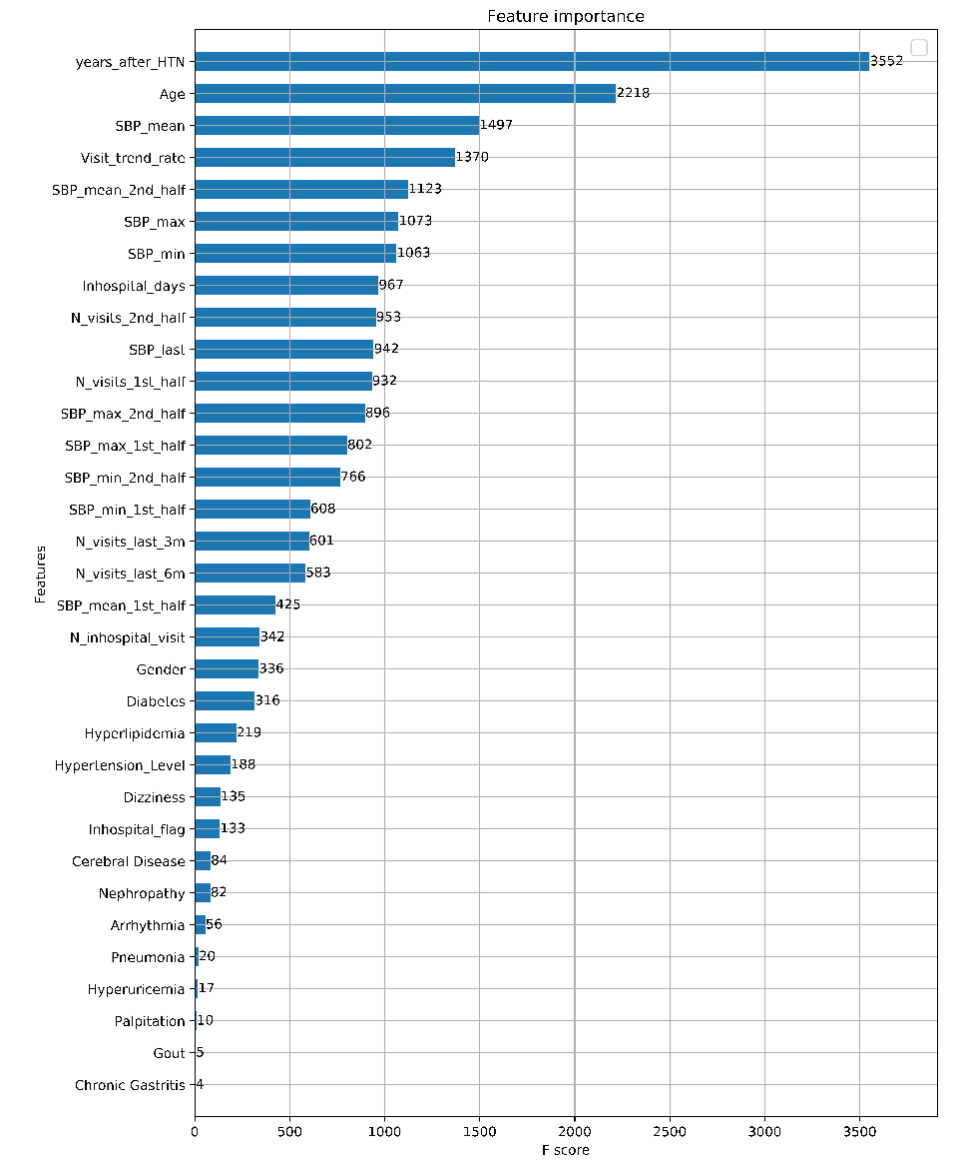
The importance rankings of feature variables for the XGBoost model.

The feature importance of the XGBoost model measures the relative contribution of the feature variables in the process of building the decision trees. Figure 3 depicts the top-ranked features selected by the XGBoost model. In addition to traditional risk factors such as age, systolic blood pressure, and years since hypertension onset, several other features representing the dynamic trends of medical activities also played important roles in risk prediction. For example, an increased frequency of medical activities (in-hospital or out-hospital visits) in the late half or the last half year of the follow-up period would be linked to a higher risk of CHD onset. In addition, the (highest or lowest) blood pressure at the late half of the follow-up period would provide additional information to the risk scores.

To further confirm the contributions of different EHR features on model precision, we performed an experiment in which a series of models were created using an increasing sequence of EHR features and the same XGBoost algorithm, and the performances of these models were tested on the same independent test set. Table 5 summarizes the variation trends of the models with different numbers of features added. Initially, variables that are traditionally used for most risk scales were selected. With only six basic variables, the model reached an AUC of 0.81, which is analogous to the performances of most of the traditional risk scales reported in the literature. Next, diagnosis variables extracted from regular follow-ups, in-hospital, or out-hospital visits were added. Although these symptom data helped to improve the model performance, the effect was quite marginal, which may be attributed to the fact that pre-CHD symptoms are mostly hidden or nonspecific and are often undiagnosed before CHD onset. Finally, variables created by combining multiple EHRs accumulated over time were added. Surprisingly, adding multiple time-point systolic blood pressure values significantly improved the accuracy of the model, suggesting that the long-term variations of blood pressure measurements can be an independent risk factor for CHD prediction. Moreover, variables indicating an increasing trend of medical activities (eg, in-hospital or out-hospital records but without a CHD-related diagnosis or medical examinations) were shown to be correlated with a future risk of CHD onset, which warrants further investigations.

**Figure 4:**
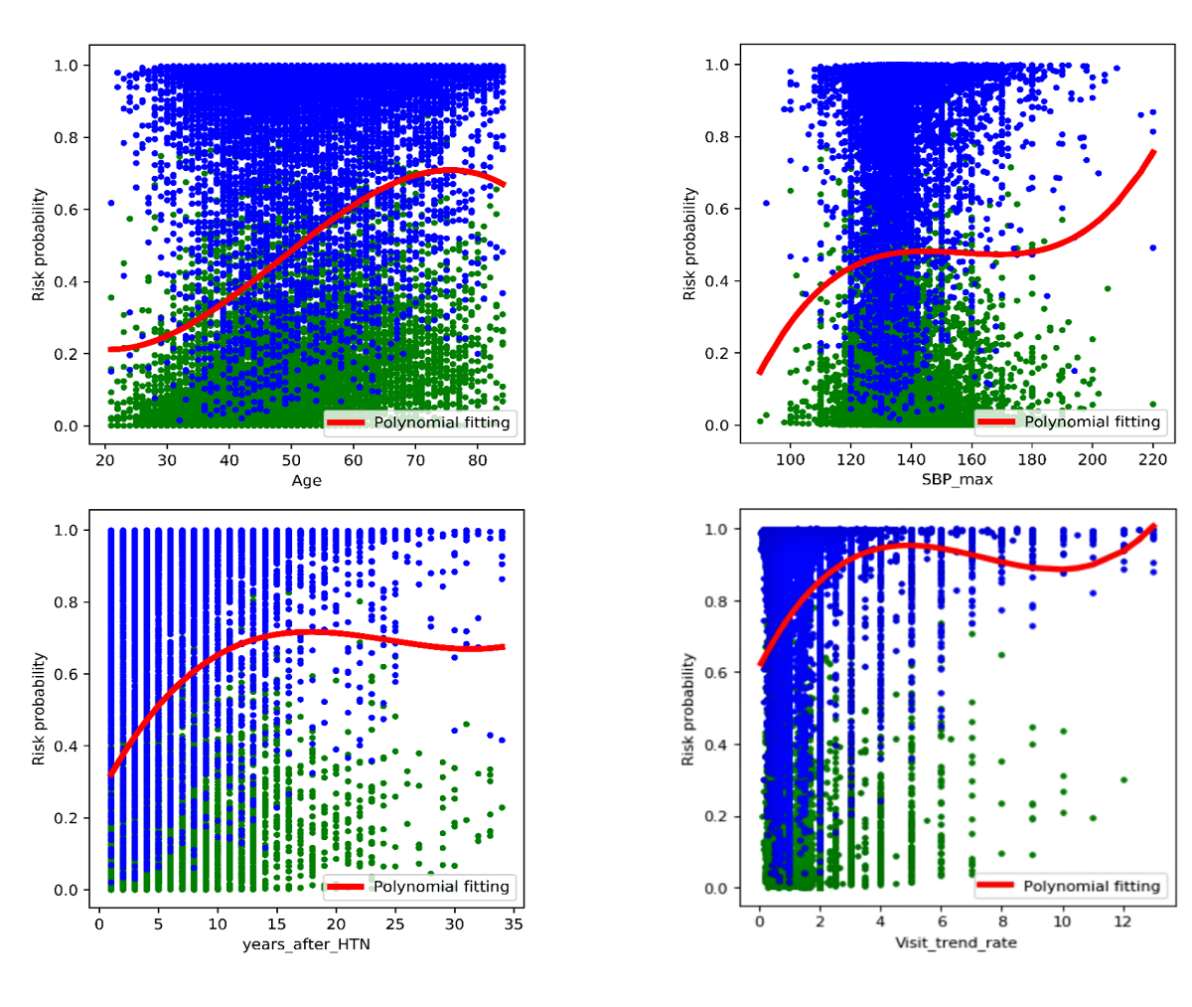
The univariate marginal effects of typical variables on the risk probability scores. The blue dots represent the CHD samples and the green dots represent the non-CHD samples in the training set. The y-axis shows the calculated risk probability scores (0=low risk, 1=high risk). The red curve shows the average risk probability at the given value/interval of the studied variables. CHD: coronary heart disease; SBP: systolic blood pressure; HTN: hypertension.

To further analyze the marginal effect of each variable, we performed a univariate trend analysis to describe the relationship between a given variable and the predicted risk probability based on the obtained model, which was visualized with a scatter plot. First, we binned all training samples (including both positive and negative samples) according to the value interval of the studied variable, which was plotted on the x-axis. The corresponding predicted risk probability for each sample was then plotted on the y-axis. Subsequently, a trend curve was plotted showing the averaged risk probability at the given value (or interval) of the studied variable. An example of the marginal effects for four typical variables is depicted in Figure 4. Many variables exhibited highly nonlinear correlations with the overall risk probability scores. This could provide useful insights for CHD prevention through improving risk factor control.

**Table 5:** Trends of model performance with increasing feature sets.

Variables included in the model	Number of features	AUC^a^	ACC^b^
SBP^c^_last, Age, Gender, Years_After_Hypertension (in the last CHD^d^-free record)	4	0.7547	0.6941
+ Diabetes diagnosis	5	0.7766	0.7090
+ Hyperlipidemia diagnosis	6	0.8111	0.7339
+Inpatient diagnosis flag +Total in-hospital days + Total in-hospital visit number	9	0.8134	0.7341
+ Diagnosed symptoms (eg, hypertension level, cerebral disease, dizziness, nephropathy, gout, hyperuricemia, palpitation)	19	0.8289	0.7460
+ Multipoint SBP statistics (SBP_max, SBP_min, SBP_mean)	22	0.8589	0.7766
+ Dynamic SBP trends (SBP_ min(max.mean)_1st(2nd)_half)	28	0.8752	0.7929
+ Medical activities trends (N_visits_1st_half, N_visits_2nd_half, Visit_trend_ratio)	31	0.9195	0.8350
+ Medical activities trends (N_visits_last_3m^e^, N_visits_last_6m^f^)	33	0.9427	0.8686

^a^AUC: area under the receiver operating characteristic curve.

^b^ACC: accuracy.

^c^SBP: systolic blood pressure.

^d^CHD: coronary heart disease.

^e^3m: 3 months.

^f^6m: 6 months.

### Impact of Population Size on Model Performance

The creation of most disease prediction models relies on large-scale research cohorts. The size of the research population is one of the important factors that affects the final performance of the created models. To determine the impact of population size on model performance, we carried out an experiment with a series of subpopulations of varying sizes and a fixed number of variables to explore the impact of different data volumes on model performance. The results are depicted in Table 6, demonstrating that the accuracy and reliability of model prediction will be improved with an increase in the size of the research population when the characteristic variables are fixed. However, given adequate variable sets, the model can reach fairly competitive performance (ie, AUC>0.8) even with a small training population size, surpassing the results obtained with a large training population but with limited feature variables (eg, Table 5). This suggested that population size is indeed a very important consideration in building disease risk prediction models but is not an overwhelming limitation.

**Table 6:** Trends of model accuracy with respect to varying training population size^a^.

Training population size (N)			ACC^b^		AUC^c^
**200**					
	Subpopulation 1	0.780		0.850	
	Subpopulation 2	0.745		0.807	
	Subpopulation 3	0.740		0.823	
	Subpopulation 4	0.770		0.840	
	Subpopulation 5	0.800		0.839	
	Mean	0.767		0.832	
**2000**					
	Subpopulation 1	0.847		0.933	
	Subpopulation 2	0.838		0.927	
	Subpopulation 3	0.833		0.921	
	Subpopulation 4	0.838		0.927	
	Subpopulation 5	0.835		0.924	
	Mean	0.838		0.926	
**20,000**					
	Subpopulation 1	0.869		0.943	
	Subpopulation 2	0.868		0.943	
	Subpopulation 3	0.869		0.943	
	Subpopulation 4	0.868		0.942	
	Subpopulation 5	0.870		0.943	
	Mean	0.869		0.943	

^a^For each size, five subpopulations were created and the results were averaged.

^b^ACC: accuracy.

^c^AUC: area under the receiver operating characteristic curve.

### Comparison With Traditional Statistical Models

Risk scales obtained by statistical analyses of relatively large samples have long been used in the prevention and screening of the high cardiovascular risk population. Several CHD risk scales have been proposed and widely adopted, such as Framingham risk scales. Therefore, it is also necessary to compare the performance of risk models obtained by machine-learning methods with these traditional risk scales. However, most existing risk scales for CVDs included lifestyle factors and blood test or medical imaging examinations that are not included in routine health checks or chronic disease follow-ups, making it hard to achieve direct comparison with EHR-based studies. In this study, we screened the cohort database to identify a subset of 536 patients (498 with CHD onset and 38 with no CHD onset) with sufficient lifestyle and blood test information required for comparison with the major existing CHD risk scales. These patients were assigned to the test dataset in the first step of our model-building process. We applied the developed XGBoost model as well as the traditional risk scales for these patients, and compared their prediction performance based on the AUC value as the evaluation metric. We should emphasize that due to the low availability in the overall population, some of the features used in the risk scales (such as smoking, diastolic blood pressure, low-density lipoprotein cholesterol, and high-density lipoprotein cholesterol) were not included in the XGBoost risk model. The following three popular risk scales were used for comparison.

#### The Framingham 10-Years CHD Risk Scale

Proposed by the Framingham Heart Study team in 1998, the Framingham 10-years CHD risk scale is now recognized as an effective tool worldwide to predict the risk and make appropriate preventive management decisions for future CHD onset at the individual level. The age range of the study population is between 30 and 74 years, and the main predictors of this simplified model include gender, age, diabetes, smoking, stratification of blood pressure (systolic and diastolic), and stratification of total cholesterol and high-density lipoprotein cholesterol [50]. It should be noted that the 10-year risk scale was designed for predicting long-term risks, which is somehow divergent from the goal of the present study. However, given that it is one of the most frequently used risk scales, we included the results for reference.

#### The Framingham 2-Years CHD Risk Scale

Proposed by the Framingham Heart Study team in 2000, the CHD 2-year risk score was developed based on the original 10-year model taking into account updated research results, further deepening and expanding models that predict the risk of recurrent or subsequent CHD events in people with a history of CHD or CVD. The age range of the model population is between 35 and 74 years. The main predictors of this simplified model include gender, age, diabetes, smoking, stratification of blood pressure (systolic), and stratification of total cholesterol and high-density lipoprotein cholesterol [4].

#### The China Multiprovincial Cohort Study Risk Scale

In 2003, based on a cohort of individuals aged 35 to 64 years living in 11 provinces and cities of China, a risk model for CVD in the Chinese population was established. This model used a prospective cohort study method to calculate the risk factors and the incidence of CVD based on predictive models. The main predictors of this simplified model include gender, age, diabetes, smoking, stratification of blood pressure (systolic), and stratification of total cholesterol and high-density lipoprotein cholesterol [51,52].

**Figure 5:**
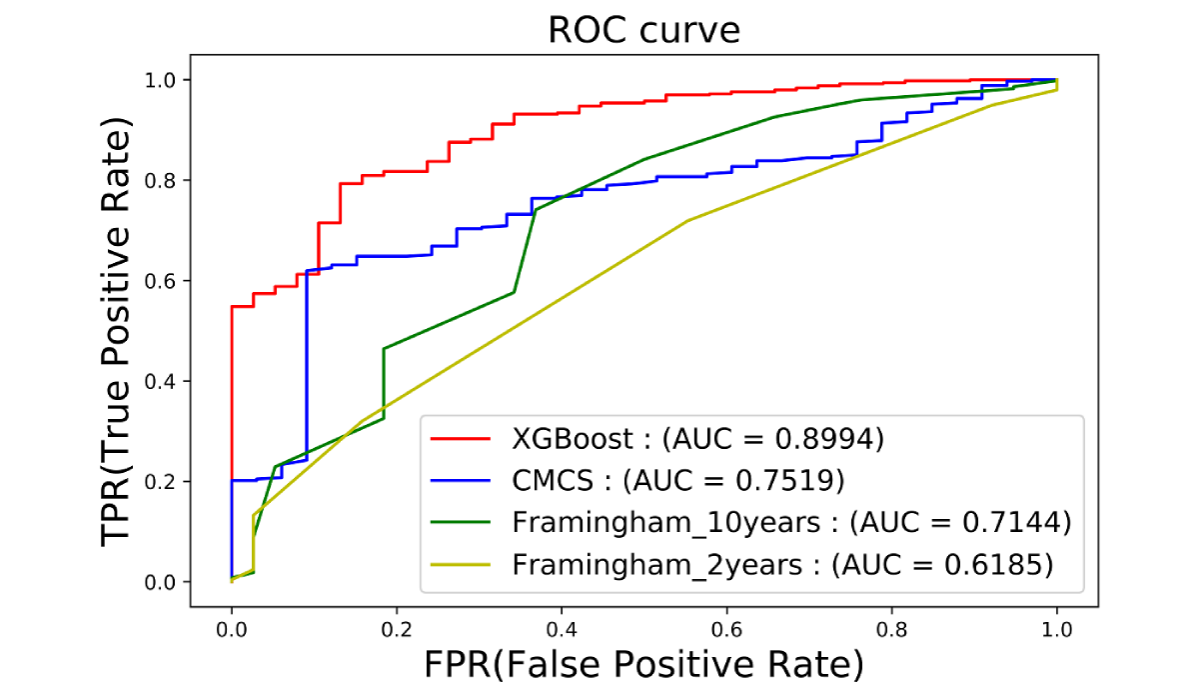
Comparison of the machine learning-based model and traditional risk scales on the same dataset.

Figure 5 shows the ROC curves achieved by the XGBoost model and traditional risk scales. The prediction model established by the XGBoost algorithm showed the best classification performance, with the AUC value reaching 0.8994, followed by the Chinese Multiprovincial Cohort Study queue model, with an AUC value of 0.7519. The prediction accuracy of the Framingham 10-year risk prediction model and 2-year risk prediction model was slightly lower, with AUC values of 0.7144 and 0.6185, respectively. Therefore, our model based on big data and machine-learning algorithms has a better classification effect, higher prediction accuracy, and better performance than traditional statistical models. Moreover, compared with traditional risk scales, our EHR-based model does not require additional medical examinations, which can reduce the patient burden and is beneficial for large-scale population screening.

## Discussion

We established a high-precision CHD prediction model through EHR big data and machine-learning techniques, and evaluated the effects of different modeling methods, the impact of feature variables, and the dataset size on the model performance. Unlike previous EHR-based studies, our model achieved high prediction accuracy (AUC=0.943) in predicting 3-year CHD onset with the independent test dataset. Further comparison analyses showed that nonlinear models outperform linear models, which was supported by the univariate marginal effect analysis showing that many feature variables had strong nonlinear effects on risk predictions.

We also demonstrated that the construction of secondary feature variables played an important role in the performances of model building. Specifically, we discovered that using time-dependent features obtained from multiple records, including both statistical variables and changing-trend variables, helped to improve the performance rather than using only static features. Moreover, with proper feather variable choices, the prediction model can achieve fairly sufficient precision even when the training sample size is small (compared with datasets from a large population but very few features). This explains the large gap of our models compared with previous EHR-based models.

In summary, our study demonstrated that accurate prediction of 3-year CHD onset risk is possible for a large group of patients with hypertension solely based on EHR data collected during routing follow-up visits for chronic diseases with in-hospital and out-hospital diagnostic records. Using an independent test dataset, we verified that EHR-based models can achieve better risk prediction performance than traditional risk scales. Compared with traditional risk scales, the EHR-based model does not involve additional medical examinations, which reduces the patient burden and is beneficial for large-scale population screening. Moreover, compared with traditional patient cohort studies, EHR-based studies are far easier to conduct with respect to data acquisition and facilitate investigating many variables in a batch simultaneously. Our results indicate that long-term accumulation of EHR big data through centralized platforms, especially the multiple time-point changes of patient health status, provides very important information for the prediction and early prevention of chronic diseases. Further investigations are needed to explore the power of accumulated historical data.

The major limitation of our study is that we used anonymized historical EHR data, which had a high missing rate. Some known potential risk factors such as diastolic blood pressure, BMI, and blood test indicators were not considered as important factors in the modeling process because of the large proportion of data missing in the population. The missing data also affected the acquisition of outcome status for each patient. The CHD onset label can be imprecise if the patient did not receive a hospital diagnosis during the study period and within the regional hospital system. This is a defect compared with traditional cohort studies. However, the impact of missing information is equal for both the positive and negative groups so that no significant biases are likely to be introduced through missing data. Compared with the benefits obtained by the enlarged population and the abundance of clinical features, the increased noise in the data is considered to be acceptable.

## Appendix

Multimedia Appendix 1 Description of fields for data collection from electronic health records.

Multimedia Appendix 2 Distribution of predicted scores in the stroke group (N=10,183).

Multimedia Appendix 3 Distribution of coronary heart disease (CHD)-free time in the CHD group (0-3 years, N=20,156).

Multimedia Appendix 4 Receiver operating characteristic (ROC) curves according to coronary heart disease (CHD) onset and follow-up times.

## Abbreviations

AUC: area under the receiver operating characteristic curve
CHD: coronary heart disease
CVD: cardiovascular disease
EHR: electronic health record
FN: false negative
FP: false positive
ICD: International Statistical Classification of Diseases and Related Health Problems
KNN: K-nearest neighbor
NPV: negative predictive value
PPV: positive predictive value
ROC: receiver operating characteristic
SVM: support vector machine
TN: true negative
TP: true positive
