# Supplementary material for: Accurate Prediction of Coronary Heart Disease for Patients With Hypertension From Electronic Health Records With Big Data and Machine-Learning Methods: Model Development and Performance Evaluation
Source: JMIR Med Inform. 2020 Jul 6;8(7):e17257. doi: 10.2196/17257 (PMC7381262; doi:10.2196/17257)
Supplement: Multimedia Appendix 1 [file medinform_v8i7e17257_app1.docx]

| **Field Names** | **Specific Signification** |
| --- | --- |
| **Residents Information Records** | |
| ID | Resident unique identification |
| SEX_CODE | Gender(Male/Female) |
| DATE_OF_BIRTH |  |
| CONFIRM_DATE | Date of confirmed hypertension |
| CHD_FLAG | Label of coronary heart disease |
| CHD_DATE | Date of confirmed coronary heart disease |
| **Regular Chronic Disease Follow-up Records** | |
| ID | Resident unique identification |
| FOLLOWUP_DATE |  |
| SYMPTOM_NAME |  |
| SBP | Systolic Blood Pressure |
| DBP | Diastolic Blood Pressure |
| HEART_RATE_TIMES |  |
| GLU | Fasting blood glucose |
| HEIGHT |  |
| WEIGHT |  |
| BMI | Body Mass Index |
| **Outpatient Diagnostic Records** | |
| ID | Resident unique identification |
| OUT_SNO | Outpatient service number |
| ADMISSIONS_TIME | Date of Outpatient service |
| DIAG_SNO | Diagnostic serial number |
| DIAG_NAME_INHOS | Name of the diagnosis |
| DIAG_CODE_INHOS | Diagnostic code(ICD-10) |
| **Outpatient Operation Records** | |
| ID | Resident unique identification |
| OUT_SNO | Outpatient service number |
| OPERATION_SNO | Operation serial number |
| OP_ARRANGE_TIME | Scheduled operation time |
| OP_NAME_INHOS | Name of the operation |
| **Outpatient Recipe Records** | |
| ID | Resident unique identification |
| OUT_SNO | Outpatient service number |
| OUT_RP_SNO | Prescription serial number |
| RP_DATE | Date of prescription |
| DRUG_NAME | Name of prescription drug |
| **Inpatient Diagnosis Records** | |
| ID | Resident unique identification |
| IN_SNO | Inpatient service number |
| IN_TIMES | Number of hospitalizations |
| ADMIT_DATE | Date of admission |
| DISCHARGE_DATE | Date of discharge |
| DIAG_SNO | Diagnostic serial number |
| DIAG_NAME | Name of the diagnosis |
| DIAG_CODE | Diagnostic code(ICD-10) |
| **Inpatient Operation Records** | |
| ID | Resident unique identification |
| IN_SNO | Inpatient service number |
| OPERATION_SNO | Operation serial number |
| OP_ARRANGE_TIME | Scheduled operation time |
| OP_NAME_INHOS | Name of the operation |
| **Inpatient Medical Order Records** | |
| ID | Resident unique identification |
| IN_SNO | Inpatient service number |
| ORDER_SNO | Medical order number |
| ORDER_BEGIN_DATE | Start date of medical order |
| ORDER_STOP_DATE | End date of medical order |
| ORDER_NAME | Name of prescription drug |
| **Biochemical test Records** | |
| ID | Resident unique identification |
| PATIENT_TYPE | Type of patient examined(outpatient/inpatient) |
| SNO | Serial number |
| LAB_SNO | Serial number for medical lab test |
| REG_DATE | Date of registration |
| LAB_TYPE_NAME | Name of medical lab test |
| ITEM_CODE | Item code for medical lab test |
| ITEM_EN_NAME | English name of medical lab test |
| ITEM_CH_NAME | Chinese name of medical lab test |
| ITEM_RESULT_DES_CODE | Tips for medical lab test |
| ITEM_RESULT_DES_NAME | Qualitative description of medical lab test |
| ITEM_RESULT_NUM | Quantitative description of medical lab test |
| ITEM_RESULT_UNIT | Unit of measurement for medical lab test |
| RESULT_REFERENCE | Reference value range |
| RESULT_REFERENCE_LOW | Lower limit of the reference range |
| RESULT_REFERENCE_HIGH | Upper limit of the reference range |
