# Supplementary figures and images for: Accurate Prediction of Coronary Heart Disease for Patients With Hypertension From Electronic Health Records With Big Data and Machine-Learning Methods: Model Development and Performance Evaluation
Source: JMIR Med Inform. 2020 Jul 6;8(7):e17257. doi: 10.2196/17257 (PMC7381262; doi:10.2196/17257)

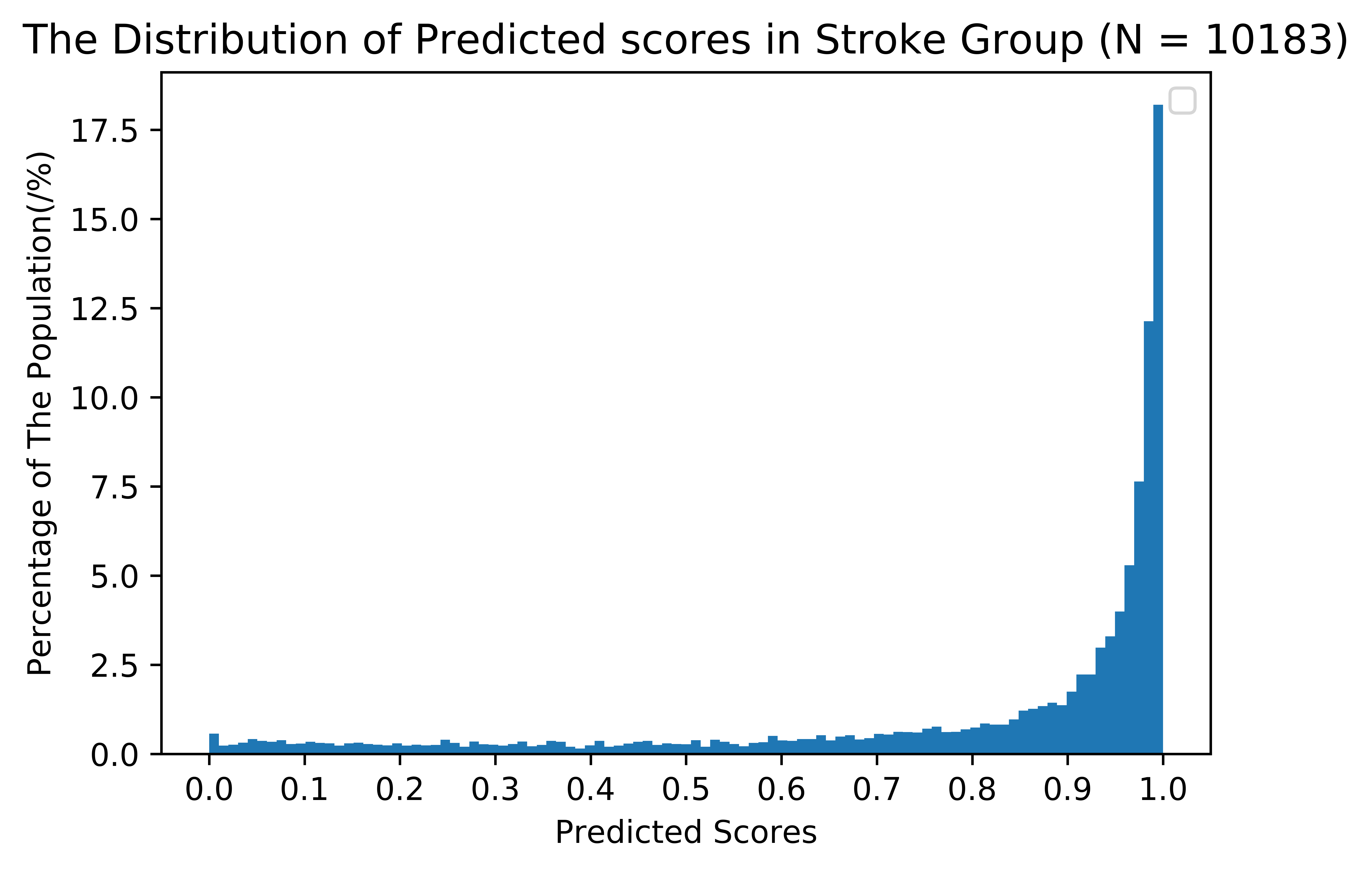

Supplement: Multimedia Appendix 2 [file medinform_v8i7e17257_app2.png]

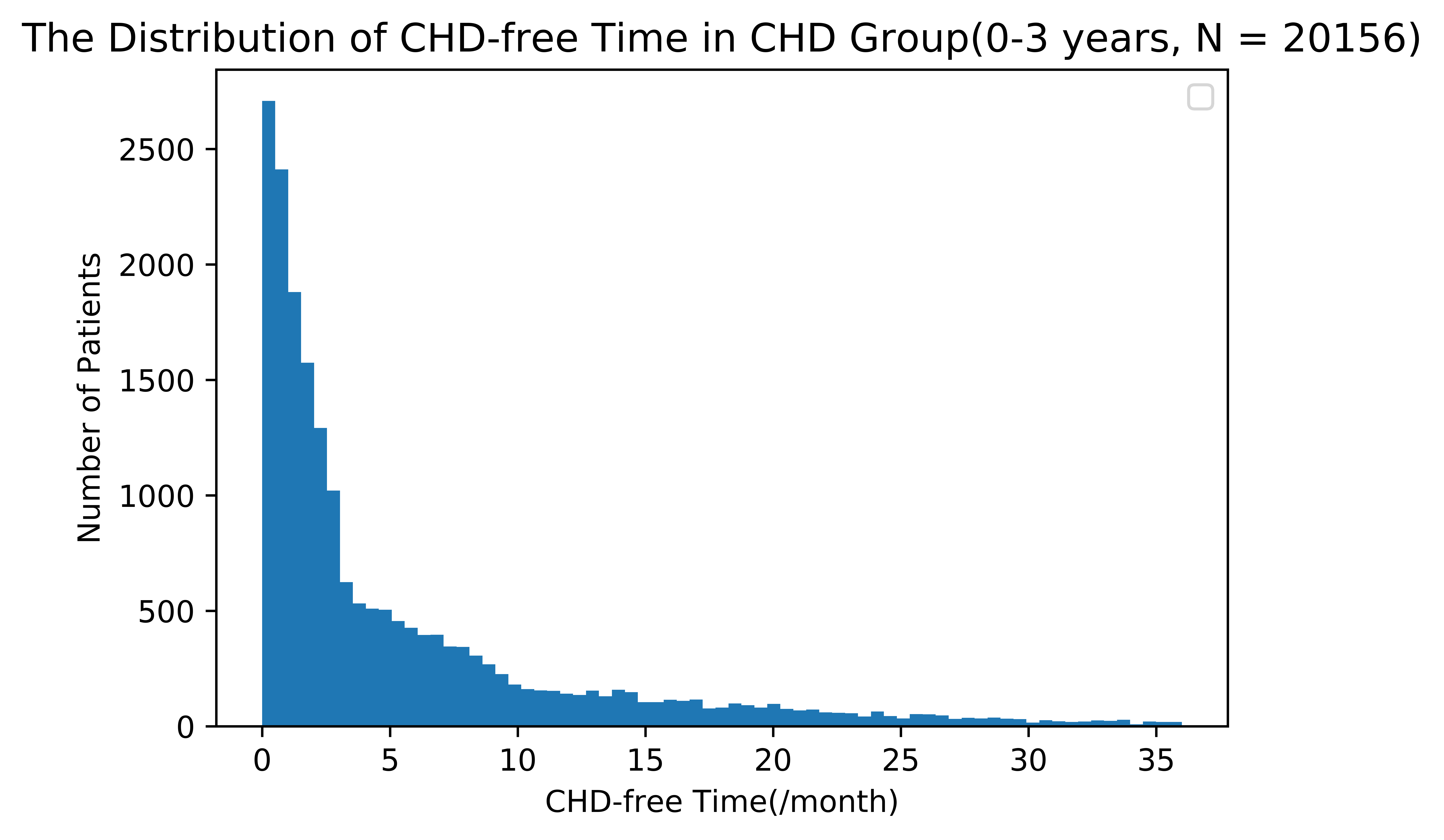

Supplement: Multimedia Appendix 3 [file medinform_v8i7e17257_app3.png]

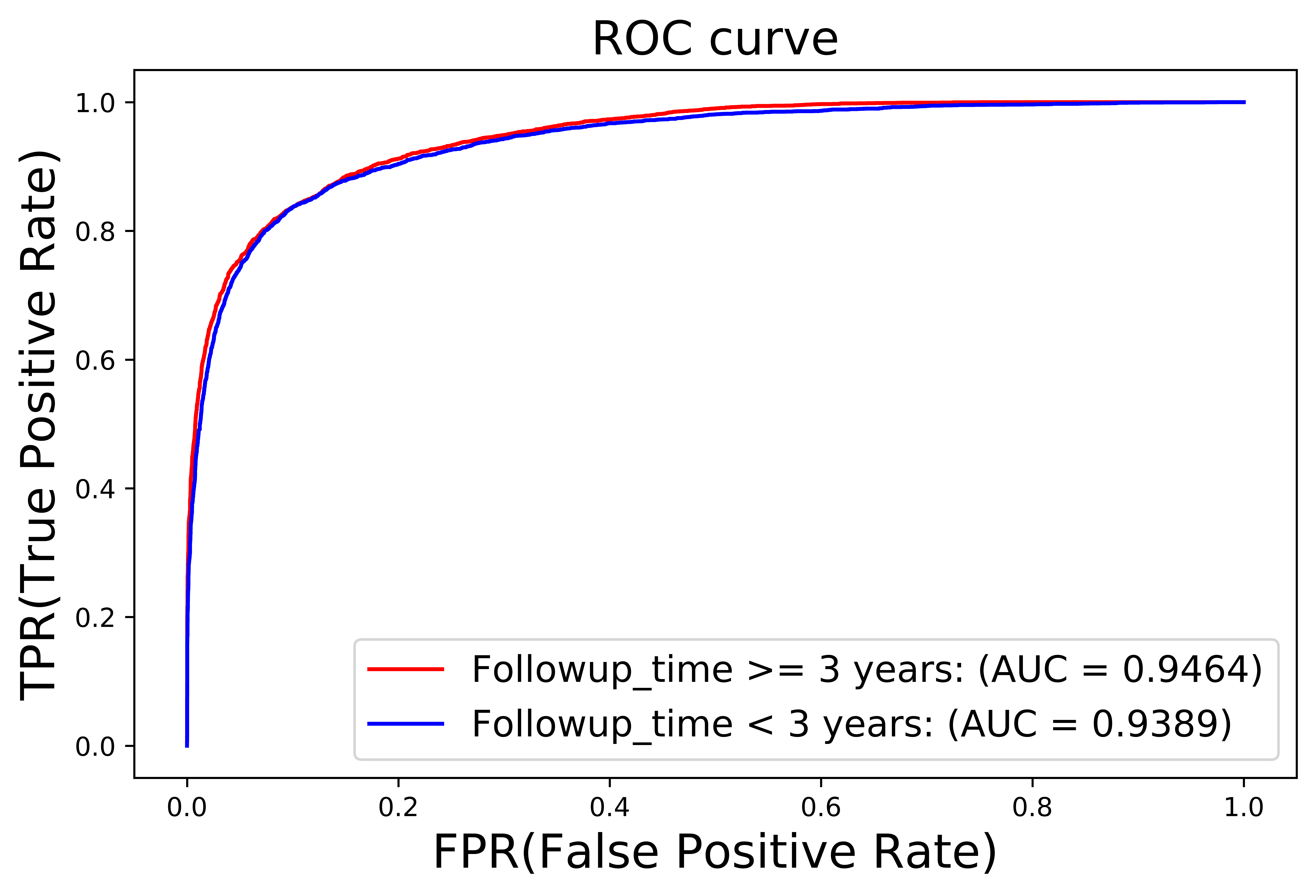

Supplement: Multimedia Appendix 4 [file medinform_v8i7e17257_app4.png]
